# Maximizing time from the constraining European Working Time Directive (EWTD): The Heidelberg New Working Time Model

**DOI:** 10.1186/s13561-014-0014-6

**Published:** 2014-09-16

**Authors:** Simon Schimmack, Ulf Hinz, Andreas Wagner, Thomas Schmidt, Hendrik Strothmann, Markus W Büchler, Hubertus Schmitz-Winnenthal

**Affiliations:** 1 University Hospital of General, Visceral and Transplantation Surgery of Heidelberg, Im Neuenheimer Feld 110, Heidelberg, 69120, Germany; 2 Biomathematician, University Hospital of General, Visceral and Transplantation Surgery of Heidelberg, Im Neuenheimer Feld 110, Heidelberg, 69120, Germany; 3 Clinic Management, University Hospital of Heidelberg, Im Neuenheimer Feld 672, Heidelberg, 69120, Germany

**Keywords:** Working time model, European working time directive, EWTD, Workload analysis, Surgical training, Heidelberg

## Abstract

**Background:**

The introduction of the European Working Time Directive (EWTD) has greatly reduced training hours of surgical residents, which translates into 30% less surgical and clinical experience. Such a dramatic drop in attendance has serious implications such compromised quality of medical care. As the surgical department of the University of Heidelberg, our goal was to establish a model that was compliant with the EWTD while avoiding reduction in quality of patient care and surgical training.

**Methods:**

We first performed workload analyses and performance statistics for all working areas of our department (operation theater, emergency room, specialized consultations, surgical wards and on-call duties) using personal interviews, time cards, medical documentation software as well as data of the financial- and personnel-controlling sector of our administration. Using that information, we specifically designed an EWTD-compatible work model and implemented it.

**Results:**

Surgical wards and operating rooms (ORs) were not compliant with the EWTD. Between 5 pm and 8 pm, three ORs were still operating two-thirds of the time. By creating an extended work shift (7:30 am-7:30 pm), we effectively reduced the workload to less than 49% from 4 pm and 8 am, allowing the combination of an eight-hour working day with a 16-hour on call duty; thus, maximizing surgical resident training and ensuring patient continuity of care while maintaining EDTW guidelines.

**Conclusion:**

A precise workload analysis is the key to success. The Heidelberg New Working Time Model provides a legal model, which, by avoiding rotating work shifts, assures quality of patient care and surgical training.

## Background

¿The individual patient, for whom we are responsible, must count on our presence and help if critical situations threaten his/her life or psyche. Such an obligation does not fit into the bounds of an 8-hour day or a 5-day week.¿ This stance about working time announced by the German surgeon, Rudolf Nissen (1896 ¿ 1981), reflects that inadequate sleep and long work hours are long-standing traditions in the medical profession.

In 1980, all doctors, including senior consultants, were present from 8 am until 6 pm Monday through Friday and junior doctors performed overnight shifts two to three times per week and throughout every second or third weekend. Depending on the rotation, junior doctors had to work and be on-call between 70 and 144 hours per week [1]. Over the years there were ongoing discussions that over-tired and inadequately supervised residents were a dangerous and weak point in patient care. Supported by milestones such as the Libby Zion case in 1984 [2], directives were developed against overwork of clinical personnel for the benefit of patients.

Derived from the European Directive of 1993 [3], the European Working Time Directive (EWTD) was initially created to improve road safety by restricting working hours of truck drivers. It laid down minimum requirements in relation to working hours, resting periods, and annual leave*.* In 2004, the EWTD provisions were extended to doctors-in-training whose maximum working hours were reduced to 56 in 2007 and 48 hours in 2009 [1]. In addition, the longest continuous shift a doctor could perform was 13 hours with an obligatory 11-hour resting time.

Because of restrictions in total time worked and duration of shift, at least three formal handovers now occur in a 24-hour period, suggesting that patients receive less continuity of care and junior doctors experience more interrupted training time. In surgical disciplines, there is evidence that work-hour restrictions do not improve patient care [4],[5]. Furthermore, because requirements for surgeons have increased due to economic pressure, more documentation tasks and higher patient load, it has become even more apparent that the EWTD cannot be maintained.

Although working hour restrictions may produce measurably happier medical trainees with better quality of life [6], it can be argued that such restrictions are detrimental in the areas of work ethic, technical skills development, and decision-making/critical-thinking skills. Moreover, disjointed shift changes and frequent handoffs come at a cost to health care delivery; lack of patient ownership cannot be disregarded [7]¿[10].

Although working hour restrictions may produce measurably happier medical trainees with better quality of life [6], it can be argued that such restrictions are detrimental in the areas of work ethic, technical skills development, and decision-making/critical-thinking skills. Moreover, disjointed shift changes and frequent handoffs come at a cost to health care delivery; lack of patient ownership cannot be disregarded [7]¿[10].

Our goal was to establish a more structured approach that balanced the consideration of both trainer and trainee¿one that provided maximum flexibility for surgeons yet was in accordance with the restrictions set forth by the European Working Time Directive.

## Methods

The New Working Time Model at the University Hospital of General, Visceral and Transplantation Surgery in Heidelberg was designed and implemented with the following strategy:

### Analysis of current status, workload, and new working time model

To evaluate the current workload, we performed statistical analyses of our department (in-house data) as well as of data from the financial- and personnel-controlling sector of our administration. Analyses of the performance data in the ORs, emergency rooms and surgical wards were done on the basis of ISHmed (SAP, Walldorf, Germany) for the time period from January to December 2007. ISHmed is a software which manages patient¿s medical documentation including diagnosis, dates of hospital stays or outpatient clinic visits, medical reports such as operation or endoscopy reports as well as results such as lab work, x-ray and cross sectional imaging pictures.

To quantify the workload of on-call duties, we used time cards that had to be completed daily by every attending (n?=?18) and resident (n?=?41) for 12 weeks between February 15^th^ and May 13^th^ of 2007, and again after implementation of the New Working Time Model from February 15th and May 13^th^ 2010. The results (workload) of this time survey were given in per cent (%): working time per presence time. An example of a 50% workload of an on-call duty is shown in Table 1.

Example of workload of an in house on-call duty (workload?=?50%)

**Table 1:** Example of a workload analysis of an on-call duty (50% workload from 4pm to 8am)

Performance	Time	Hours working time	Hours resting time
Operation	16:00 ¿ 20:30	4,5	
Resting time	20:30 ¿ 0:30		4
Emergency room	0:30 ¿ 3:00	2,5	
Resting time	3:00 ¿ 7:00		4
Handover/morning meeting	7:00 ¿ 8:00	1	
		8	8

To quantify the time of doctor performances we hired an independent health care company which personnel accompanied surgical attendings and residents from December 2007 until March 2008.

To determine how many doctors were required for providing medical care of consistent quality (manpower requirement), we used the formula ¿required working time¿ divided by 1780.8 working hours (106,848 minutes), which were defined by the personnel-controlling sector of our administration as the clear working time of a full-time doctor per year.

Time constraints for research and teaching were ascertained through personal interviews and evaluated from teaching schedules of the surgical department.

Three new working time models were designed, taking into account the calculated workload in different areas of the clinic. All proposed changes met the requirements of the EWTD.

### Proposal

a. Presentation of the current status analyses and of the three prospective new working time models to:

i. All surgeons

ii. Middle and top management

iii. Work Council

b. Identification of the best model and permission for its implementation

c. Detailed preparation of involved staff

### Implementation

The new model was implemented on October 1st 2009 for all surgeons working in the identified areas for a trial period of six months. After six months, a formal evaluation was performed in order to validate the New Working Time Model. The results of this analysis were presented to:

i. All surgeons

ii. Middle and top management

iii. Work Council

After permission, the model was implemented permanently. All data were collected in compliance with the Helsinki Declaration.

## Results

### Workload and manpower requirement prior to the new working time model

At the University Hospital of General, Visceral and Transplantation Surgery, one of the leading surgical departments in Germany, 60 surgeons worked on seven wards with 132 beds, including an intermediate care (IMC) unit and an intensive care unit (ICU), each with 15 and 14 beds, respectively.

During the analysis period between January 1^st^ and December 31^st^, 2007, 10,792 operations and procedures were performed in nine different operating rooms (central theater?=?6, emergency room?=?3). As shown in Table 2, 25,371 patients were treated in the emergency room, 22,690 patients were seen by 11 various specialized consultations, and 8,253 endoscopies were performed.

**Table 2:** Current status analysis of 2007: emergency room and outpatient clinic

	**Patients treated**	**Per day**	**Full time doctor per performance**	**Time [min] per performance**	**Working time required [min]**	**Manpower requirement**
**Emergency room**	25,371	70	1.2	21	639,349	5.99
**Outpatient operations**	6,795	27	1.5	40	407,700	3.82
**Endoscopies**	8,253	33	0.5	35	144,428	1.35
**Consultations attendings**	1,377	6	1	20	27,540	0.26
**Consultation private patients**	1,968	8	1	20	39,360	0.37
**Consultation oncology**	1,238	5	1	30	37,140	0.35
**Consultation gastroenterology**	2,449	10	1	40	97,960	0.92
**Consultation liver**	1,240	5	1	20	24,800	0.23
**Consultation kidney**	700	3	1	35	24,500	0.23
**Consultation proctology**	411	2	1	45	18,495	0.17
**Consultation thyroid gland**	300	1	1	20	6,000	0.06
**Consultation traumatology**	2,928	12	1	30	87,840	0.82
**Consultation hand**	3,816	15	1	15	57,240	0.54
**Consultation wounds**	6,263	25	1.2	20	150,312	1.41
**Resusitation room**	355	2	1	60	21,300	0.20
**Plaster room**	4,214	17	1	3	12,642	0.12
**In total 2007**	**67,678**	**241**			**1,796,606**	**16.82**

In 2007, our clinic taught 300 students using five different modules, which included lectures, seminars, medical-skills labs, bedside teaching as well as interdisciplinary readings. On average, each physician taught for two hours per week. Teaching activities were counted as full-time work and were organized by the section of the Heidelberger Curriculum Medicinale (HeiCuMed). All research activities, as part of the duty of academic surgeons, were organized and performed independently by each surgeon and were inclusively added to the working time with 4.8 hours per week (10%) as fixed in the contract.

To determine the current status of our clinic and to calculate the required manpower for the New Working Time Model, we analyzed the medical performance data of our hospital as well as every doctor¿s input and total time. Three areas of patient care were identified as being relevant: operating room, emergency room/outpatient clinic, and wards.

### Operation theater

In 2007, we performed 3,997 operations in our central operating room (Table 3). On weekdays, an average of 14.6 (13.8-14.9) operations was performed per 24 hours. On Saturdays, Sundays, and holidays, approximately 3.5 operations per 24 hours were documented. In total, an average of 3.3 surgeons was involved in each operation with an average operation time of 145 minutes. Due to educational purposes, one attending, one surgical fellow and at least one surgical resident were present at each operation.

**Table 3:** Current status analysis of 2007: central operation theater

	**Number of operations**	**Full time doctor per performance**	**Time [min] per operation**	**Working time required [min]**	**Manpower requirement**
**Operations**	3,997	3.3	145	1,912,565	17.86
**Setup-time**	3,997	2	60	479,640	4.48
**Waiting time (2.5%)**				47,814	0.45
**Transferring patients to recovery room**	3,997	1	5	19,985	0.19
				**2,460,004**	**22.98**
		**Operations**		**Per day**	
**Monday**		691		13.8	
**Tuesday**		745		14.9	
**Wednesday**		724		14.5	
**Thursday**		718		14.7	
**Friday**		759		14.9	
**Saturday**		178		3.5	
**Sunday**		182		3.5	
		**3997**			

**Figure 1:**
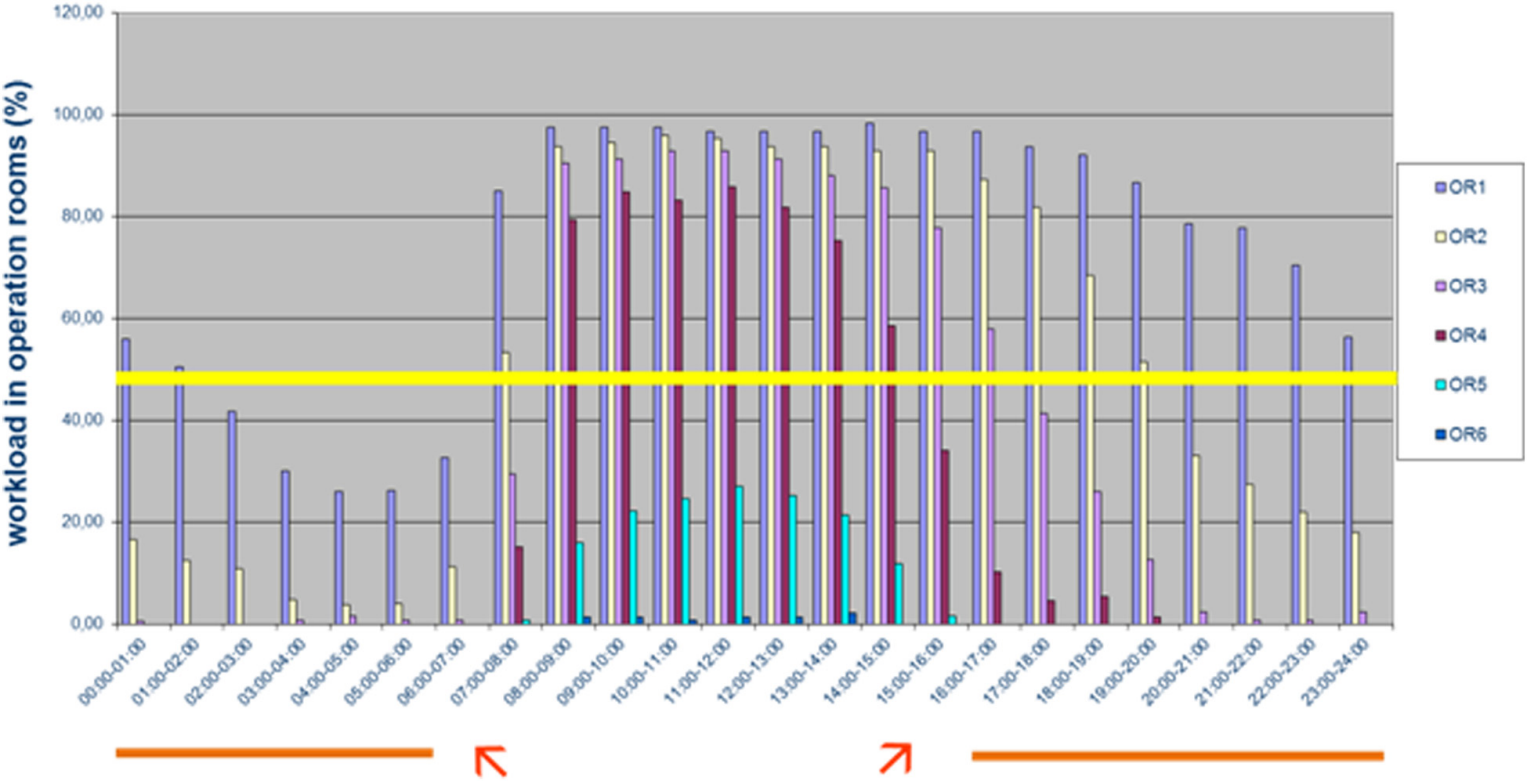
**Working load analysis of operation theater.** Operating rooms (ORs) that were running at full-time (or greater than 49% workload) during various time shifts: between 8 am and 4 pm, four ORs were running; until 8 pm, two; and until 2 am, one. The yellow line designates the critical 49% workload, which cannot be exceeded for more than 9.25 hours in a 24-hour period. The orange line denotes the workday that extends beyond the 13-hour EDTW guideline. The red arrows indicate the 9.25-hour period of full-time work.

All operations in the central operating room, including setup and transfer time, required 2,460,004 minutes or 41,000.1 hours of physician time (excluding anesthesiologists), which equated to 22.98 full-time doctor positions per year (Table 3). The workload analysis over regular working days indicated that four ORs were running at full-time (or more than 49%) from 8 am until 3 pm; three, from 3 pm until 5 pm; two, from 5 pm until 8 pm; and one, from 8 pm until 2 am. Between 2 am and 8 am the average workload in the operation theater was below 49%, or less than full-time. Figure 1 displays the number of ORs in use at full-time within a 24-hour period during weekdays.

### Emergency room and outpatient clinic

In 2007, 67,678 patients were treated in the emergency room and specialized consultations (Table 2). 25,371 patients required the help of emergency room physicians, averaging 70 patients per 24 hours and totaling 639,349 working minutes or 10,655.81 working hours. We performed 6,795 interventions and operations in three emergency ORs, which produced an average of 27 procedures per day and 407,700 minutes or 6,795 hours working time (Table 2). All together, we worked 1,796,606 minutes or 29,943.43 hours in the emergency room during 2007, which equated to the use of 16.82 full-time doctors.

**Figure 2:**
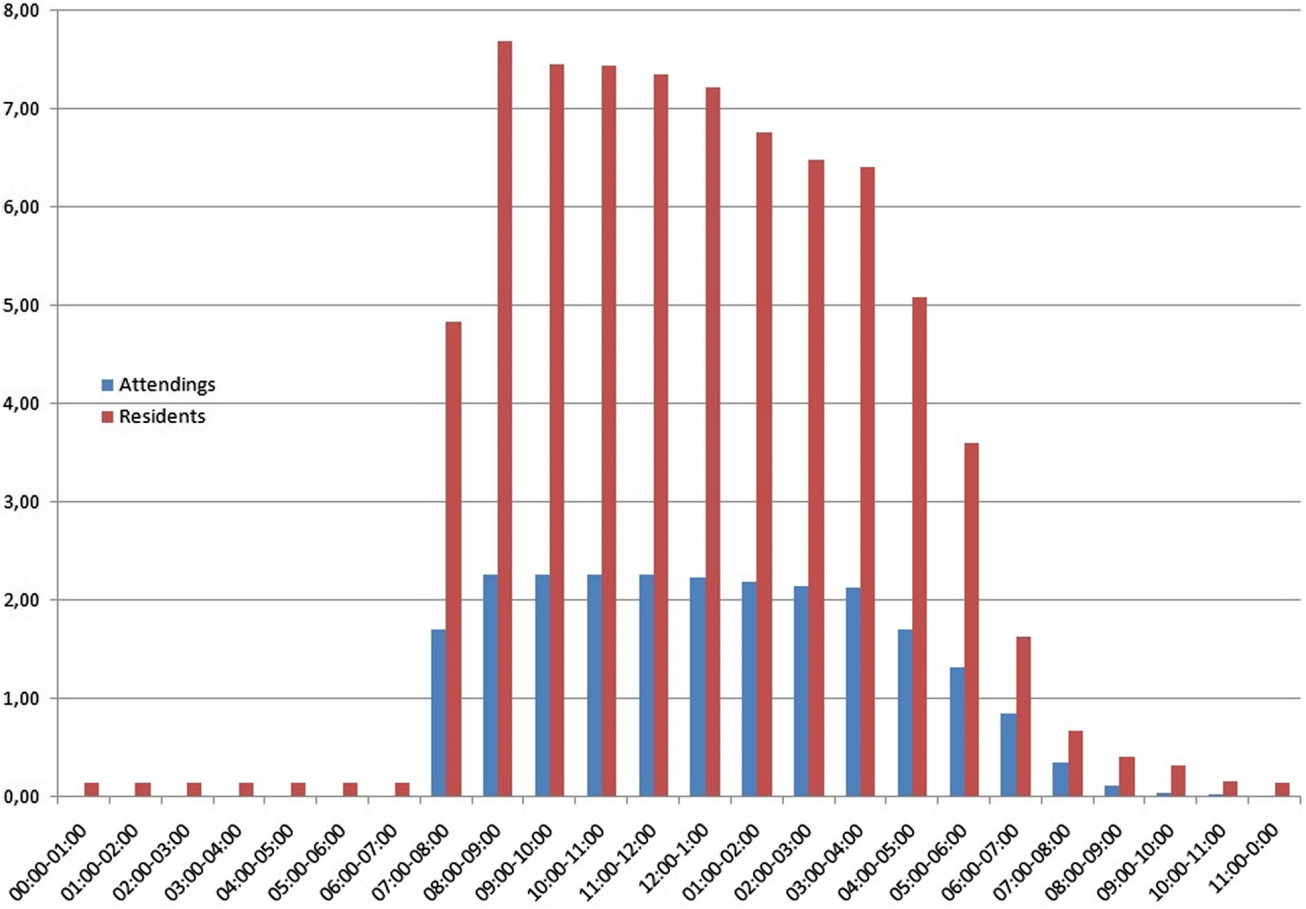
Presence of emergency room surgeons on working days in 2007.

In general, there were two attendings and 7 residents working from 8 am-2 pm, and 5 residents from 7 am-5 pm (Figure 2). After 6 pm, emergency room doctors were supported by 4 residents on 24-hour service, who were responsible from 7:30 pm onward.

### Surgical wards

In 2007, 7,406 patients were treated on five different wards (providing 103 beds). The average stay of our patients was 6.4 days, which totaled to 47,682 days of inpatient caretaking (Table 4). In Table 5, we demonstrate the analyzed workload of ward 8 to exemplify ward work, in general. In ward 8, 1,364 patients were managed over 11,156 days. All treatments, such as ward rounds, administration and computer work, as well as caretaking and talking to family members, were performed in 418,884 minutes or 6,981.4 hours by a calculated 3.92 full-time doctors.

**Table 4:** Current status analysis of 2007: inpatient care taking

	**Beds**	**Patients**	**Days**
**Ward 1**	**16**	**833**	**4,766**
**Ward 2**	**16**	**883**	**4,644**
**Ward 3**	**15**	**614**	**4,639**
**Ward 5**	**21**	**1,047**	**6,377**
**Ward 8**	**35**	**1,364**	**11,156**
**IMC**	**14**	**1,721**	**4,900**
**ICU**	**15**	**771**	**5,214**
**Total**	**132**	**7,406**	**47,682**

**Table 5:** Current status analysis of 2007: normal ward

**Ward 8: tasks**	**Patients/days**	**Full time doctor per performance**	**Time [min] per performance**	**Working time required [min]**	**Manpower requirement**
**Initial treatment**	1,364	1	30	40,920	0.38
**Discharge**	1,364	1	20	27,280	0.26
**Talking to family members**	1,364	1	17	23,188	0.22
**Medical report**	1,364	1	23	31,372	0.29
**Computer work**	1,364	1	12	16,368	0.15
**Care taking**	11,156	1	11	122,716	1.15
**Administration**	11,156	1	5	55,780	0.52
**Additional effort for isolated patients**	712	1	15	10,680	0.10
**Morning ward round**	250	1	122	30,500	0.29
**Weekend ward round**	115	1	92	10,580	0.10
**Attending ward round (2/week)**	100	2	92	18,400	0.17
**Chief ward round (1/week)**	50	2	92	9,200	0.09
**Others**	365	2	30	21,900	0.21
**In total**				**418,884**	**3.92**

The intermediate and intensive care units were analyzed separately. Analyses of the IMC are demonstrated in Table 6 as general example for intensive care workload. Due to high workload, doctors on IMC and ICU worked according to a two-shifts model (13 hours/day: 7 am-8 pm; 13 hours/night: 7 pm-8 am [1 hour handover]; 11 hours resting time in between; maximum 4 shifts in a row).

**Table 6:** Current status analysis of 2007: Intermediate Care Unit (IMC)

**IMC/VTS: tasks**	**Patients/days**	**Full time doctor per performance**	**Time [min] per performance**	**Working time required [min]**	**Manpower requirement**
**Initial treatment**	1,721	1	45	77,445	0.73
**Patient transfer/discharge**	1,721	1	30	51,630	0.48
**Medical report**	1,721	1	23	39,583	0.37
**Computer work**	1,721	1	12	20,652	0.19
**Intensive care**	4,900	1	45	220,500	2.06
**Communication/organisation**	365	1	65	23,725	0.22
**Hand-over Mon-Fri**	250	2	60	30,000	0.28
**Hand-over Sat, Sun & holidays**	115	2	60	13,800	0.13
**Talking to primary physicians/family**	365	1	80	29,200	0.27
**Administration**	4900	1	5	24,500	0.23
**Morning ward round**	250	2	40	20,000	0.19
**Weekend morning ward round**	115	1	40	4,600	0.04
**Noon ward round**	200	2	54	21,600	0.20
**Night ward round**	365	1	27	9,855	0.09
**Case reports**	50	2	54	5,400	0.05
**Coordination (performed transplantation)**	263	1	90	23,670	0.22
**Coordination (canceled transplantation)**	250	1	60	15,000	0.14
**Contact transplantation office**	50	1	45	2,250	0.02
**Patient transfer/diagnostic**	200	1	45	9,000	0.08
**Additional effort for isolated patients**	624	1	20	12,480	0.12
**Equipment instructions**	12	6	60	4,320	0.04
**Others**	365	2	30	21,900	0.21
**In total**				**681,110**	**6.38**

### Night shifts

During nights, there was one attending, one fellow, and four residents responsible for the emergency room, general wards, and the OR. The OR ran two rooms, plus one room that could be available within 60 minutes, if necessary. On-call duties started at 7:15 am and ended at 8 am the following day. Work from 7:15 am until 3:50 pm was considered standard work time, whereas 3:51 pm until 8 am was designated as on-call duty.

During nights, three additional surgical residents worked on ICU and IMC as well as one transplantation/explantation team (three surgeons for liver, kidney and pancreas transplantation) and one lead attending on-call.

### The Heidelberg new working time model

In order to maintain high quality patient care and to minimize errors due to frequent shift changes, our goal was to establish an EWTD-compliant New Working Time Model that minimized handovers and provided maximal flexibility regarding working time for surgeons. Secondly, we wanted to exploit the EWTD maximum allowable working time in order to provide the best care possible for patients and to enable surgeons in training to gain adequate experience in a reasonable time period.

To meet these criteria, we preferred a 24-hour based system over a shift model. All surgeons, the clinic board, and the work council fully supported this idea. According to the EWTD, the average workload over 49% should be limited to eight hours followed by a 16-hour working period with an average workload of less than 49%.

Two main problems according to our analysis needed to be addressed:

1. The required working time of all areas of patient care and patient management (6,856,610 minutes or 114,276.8 hours/year) was higher than the product of employed full-time surgeons and working hours per surgeon per year (60 × 106,848?=?6,410,880 minutes or 106,848 hours/year). To solve this problem and to cover those 445,730 minutes or 7,428.8 hours, we employed 4 additional surgical residents.

2. The detailed workload analysis indicated a need for workload reduction of the on-call team between 4 pm and 8 am to meet EWTD requirements since the workload of this time period was around 80% (*data not shown*), which caused an average workload of more than 49% between 3.51 pm and 8 am. The New Working Time Model addressed this topic by introducing a long, full-time day shift from 7:30 am until 7:30 pm, thereby reducing the workload of the in-house on-call team to less than 49% by covering the time from 3:51 pm to 7:30 pm.

**Figure 3:**
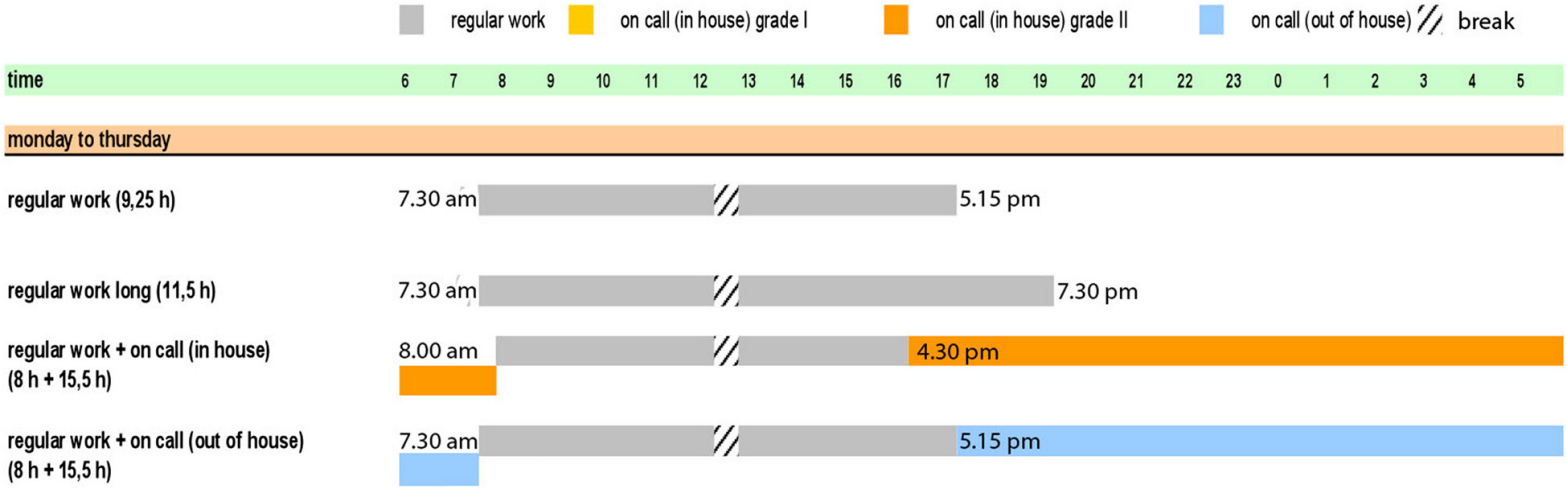
Heidelberg New Working Time Model (work days), implemented since October 2009.

The New Working Time Model is demonstrated in Figure 3. This model, as well as two alternatives supporting a 12- or 9-hour work shift, was presented to all surgeons of the department, middle and top management, and work council. The 24-hour model was chosen as the most convenient model and its implementation was prepared as described in Methods.

### After implementation of the new working time model

**Figure 4:**
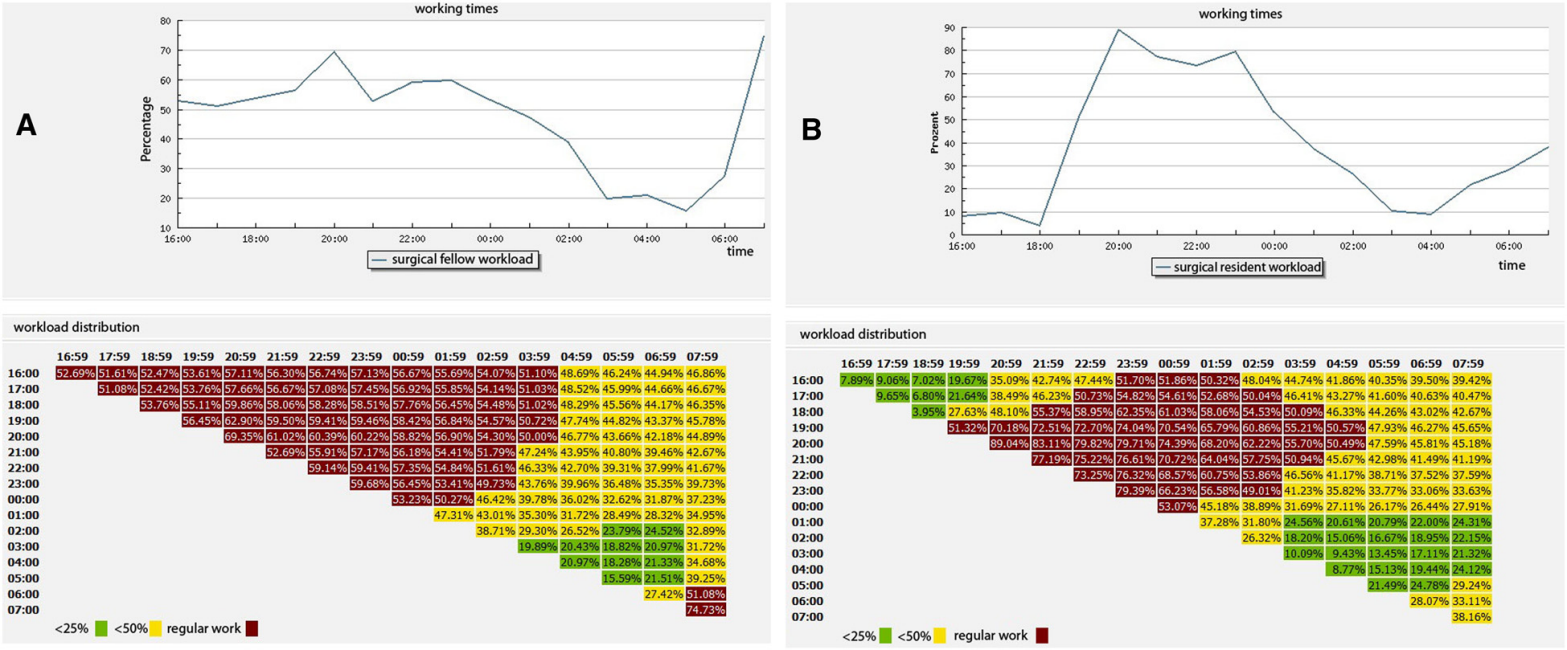
**Two examples of workload analyses after the implementation of the New Working Time Model. A**: Surgical Fellow, **B**: Surgical Resident (emergency room). In total, the workload from 16:00 (4 pm) to 08:00 (8 am) was below 49% for both, thus giving the opportunity to combine a regular full-time working day with a 15.5-hour on-call duty. (X-axis: European time; y-axis: percent of work; Workload green: <25%; yellow: <50%; red: regular work >50%. This graph was created by the online software of ww2.gob-tauch.de.

The new model was implemented in October 1^st^ 2009. After an implementation phase of six months, we specifically re-analyzed the workload from 4 pm to 8 am by using time cards from February 15^th^ to May 13^th^ 2010. The results are shown in Figure 4. The extended work shifts (7:30 am-7:30 pm) reduced the workload to less than 49% between 4 pm and 8 am; thus, allowing the combination of an eight-hour working day with a 16-hour on-call duty, a schedule that complied with EDTW standards and effectively avoided a shifts model.

## Discussion

This work presents firstly, a new working time model, which allows continuous patient care, avoids many handovers and ¿shift-worker-mentality,¿ and complies with the European Working Time Directive. Secondly, and most importantly, it provides the methods to develop a suitable working time model. The key to success of the New Working Time Model was the detailed and careful analysis of doctors¿ work and workload in all working areas of the hospital, as demonstrated in Tables 2, 3, 4, 5 and 6.

The central operation room revealed to be the working area that was most costly in terms of labor (2,460,004 minutes or 41,000.1 hours in 2007). It was remarkable that 547,439 minutes were required for setup and waiting time alone, which necessitated the use of five full-time surgeons. The most important finding for the design of the New Working Time Model was the discovery that two ORs were still utilized until 8 pm (Figure 1). The time from 4:30 pm until 8 pm could not be covered by the on-call team. Therefore, we established an extended day shift (7:30 am until 7:30 pm). This team consisted of two attendings, two fellows, and four residents, which effectively reduced the workload of the on-call team to below 49% (Figure 4).

A three-shifts-a-day model (9-hour shifts, each with one-hour handover) is a simple, and often used model by the EWTD; however, it does not address the uniqueneeds of medical care at all [11]. Our model is an attempt to protect surgeons¿ needs for adequate education and operation time, to ensure quality of patient care, and to allow a stable mentor-trainee and doctor-patient relationship within EWTD boundaries.

Compared to other medical fields, surgery, as a craft specialty, is more affected by time restrictions set by the EWTD. Since its inception, the EWTD has considerably reduced resident attendance from 225 days per year to 150¿160 days per year. As a consequence, trainees perform fewer procedures because of absent time during enforced resting periods (30% less operations per year) [9],[12],[13]. Furthermore, long operations are not work-shift compatible as team changes during operations are dangerous. The reduction in shift lengths necessarily leads to more transitions of patient care responsibility from one physician to another and, consequently, to a loss in ¿patient ownership thinking¿ [10] as well as to an increase in administrative duties [14]. Therefore, every European hospital is called to perform a similar workload analysis in order determine the possibility to design a more sufficient working time model and to avoid work shifts and their disadvantages.

On the other hand, concern about negative effects of sleep deprivation on residents¿ well-being is one of several factors behind the mandatory EWTD and is therefore applauded by sleep researchers [15]. This pertains especially to resident physicians whose work schedules are notoriously intense. To function well, humans require adequate sleep. The original goal for proposing work hour limits was to increase the rest time of residents, thereby improving their safety and quality of life [16],[17], as well as reducing the incidence of fatigue-related medical errors in an effort to improve patient safety [18],[19]. Bohrer et al. investigated quality of life of surgeons in Germany in a large scale study and discovered that 68% of surgeons, as opposed to only 39% of non-surgeons, worked more than 60 hours per week on average [20]. Compared to non-surgeons, surgeons reported more restrictions on their private and family life due to work overload (59% vs. 74%) [20]. This was confirmed by Fletcher et al., who showed that work hour restrictions improve quality of life by allowing more time for family events and decreasing fatigue, in general [9].

However, our study shows that the EWTD greatly impacts the volume and quality of surgical training, suggesting that the education of competent and experienced surgeons may no longer be feasible in the standard six-year surgical residency. Moreover, no study has ever evaluated the effect of work hour reductions on the performance of physicians after the completion of training [11].

In Heidelberg, *academic* surgeons must be competent surgeons and research scientists; therefore, surgical experience and research training needs to be guaranteed. This ambitious trajectory is unlikely to be accomplished following EWTD guidelines. Additionally, the time restrictions will undoubtedly have adverse effects on scientific research. As serious scientific work appears to be very difficult within EWTD limits, medical research becomes no longer internationally competitive. Consequently, this leads to unpaid ¿voluntary¿ scientific work during resting times by ambitious surgeons and, even worse, to a deployment of some of Europe¿s most talented physicians to other countries, such as USA or Canada [7].

At this point, questions arise as to whether such a career path of academic surgeons is still up-to-date in the time of the ¿Millennial generation¿, a demographic known to place greater importance on having a flexible schedule with adequate work/life balance and less on older concepts of success such as ¿no gratification without suffering¿ with the promise for substantial compensation later on (http://www.pwc.com/gx/en/hr-management-services/publications/nextgen-study.jhtml).

## Conclusion

We, at the University Hospital of General, Visceral and Transplantation Surgery in Heidelberg and as a very competitive center, have produced a New Working Time Model in response to the European Working Time Directive that avoids shift work and provides as much as possible, continuous patient care and sufficient surgical education. The key for every hospital or medical department to adapt their working time model to the European Working Time Directive and their changing needs is an extensive and precise analysis of the workload.

## Competing interests

The authors declare that they have no competing interests.

## Authors contributions

SS participated in acquisition, analysis and interpretation of data and drafted the manuscript, UH proved all numbers and carried out statistical analyses, AW contributed to study conception, data acquisition and manuscript drafting, TS and HS helped to draft the manuscript and to design the figures, MWB participated in study design and coordination and revised the manuscript, HSW is responsible for study design, interpretation of data and revised the manuscript.
